# Palm-Vein Classification Based on Principal Orientation Features

**DOI:** 10.1371/journal.pone.0112429

**Published:** 2014-11-10

**Authors:** Yujia Zhou, Yaqin Liu, Qianjin Feng, Feng Yang, Jing Huang, Yixiao Nie

**Affiliations:** 1 School of biomedical engineering, Southern Medical University, Guangzhou 510515, China; 2 Department of Electrical and Computer Engineering, University of Illinois at Urbana-Champaign, Champaign, 61820, United States of America; The University of Science and Technology of China, China

## Abstract

Personal recognition using palm–vein patterns has emerged as a promising alternative for human recognition because of its uniqueness, stability, live body identification, flexibility, and difficulty to cheat. With the expanding application of palm–vein pattern recognition, the corresponding growth of the database has resulted in a long response time. To shorten the response time of identification, this paper proposes a simple and useful classification for palm–vein identification based on principal direction features. In the registration process, the Gaussian-Radon transform is adopted to extract the orientation matrix and then compute the principal direction of a palm–vein image based on the orientation matrix. The database can be classified into six bins based on the value of the principal direction. In the identification process, the principal direction of the test sample is first extracted to ascertain the corresponding bin. One-by-one matching with the training samples is then performed in the bin. To improve recognition efficiency while maintaining better recognition accuracy, two neighborhood bins of the corresponding bin are continuously searched to identify the input palm–vein image. Evaluation experiments are conducted on three different databases, namely, PolyU, CASIA, and the database of this study. Experimental results show that the searching range of one test sample in PolyU, CASIA and our database by the proposed method for palm–vein identification can be reduced to 14.29%, 14.50%, and 14.28%, with retrieval accuracy of 96.67%, 96.00%, and 97.71%, respectively. With 10,000 training samples in the database, the execution time of the identification process by the traditional method is 18.56 s, while that by the proposed approach is 3.16 s. The experimental results confirm that the proposed approach is more efficient than the traditional method, especially for a large database.

## Introduction

The development and popularity of computers and the Internet, particularly electronic commerce, have rendered biometrics-based automated human identification as very important and indispensable [1]. Vein recognition is an automated human identification technology based on the vein pattern, which is the vast network of blood vessels under human hand skin [2]–[5]. Compared with other biometrics technology, such as that using fingerprints [6], [7], palmprints [8]–[10], [26], and iris [11], palm–vein recognition [5], [39], [40] has the advantage of uniqueness and abundance of identity information, live body identification, counterfeiting difficulties, etc. These advantages confirm palm–vein recognition as a promising and effective technology with the merits of high accuracy and wide application range.

Existing palm-vein identification algorithms focus on improving the accuracy of one-to-one matching, which can be broadly categorized in three categories: (1) subspace learning approaches [13], [14] such as locality preserving projection (LPP) in [13] and scale invariant feature transform (SIFT) in [14], (2) statistics-based methods [15]–[17] such as the image-invariant moment [15], [16] and local binary pattern (LBP) and its variant local derivative pattern (LDP) [17], (3) texture-based coding [12], [18]–[25] such as Radon transform [12], Gaussian function and its variants [18]–[21], and Gabor-based methods [22]–[25]. Among various palm–vein matching schemes, orientation-based coding, as a valid representation of palm–vein patterns, has the advantages of high accuracy, robust illumination, and fast implementation.

The algorithms described in the literature are designed for more delicate one-to-one comparisons in palm–vein verification, most with the accuracy of more than 95% and the response time within 1 s, which basically conform to the real-time applications of palm–vein verification. When used in one-to-many applications of palm–vein identification, the input palm–vein pattern is matched with all the palm–vein patterns in a database. If the database is very large, real-time requirements may not be fulfilled. For example, the Gabor-based methods in [18] have the shortest response time of 0.70 ms for one matching. For a 10^6^ palm–vein database, the response time of the identification was nearly 11.67 min. To shorten the response time of palm–vein identification, palm–vein images should be classified based on certain features. Palm–vein images can be assigned into some bins rather than into only one bin. The input palm–vein pattern must be matched only by a subset of palm–vein patterns in the entire database. Literature claims that problems of fingerprint classification [27], [28], [29] and palmprint classification [30], [31] have been addressed, but studies remain limited in the area of palm–vein identification. Besides, approaches proposed for fingerprints or palmprints are difficult to adopt for palm–vein recognition because of particular characteristics of palm–vein, such as irregular texture structures and low contrast.

**Figure 1:**
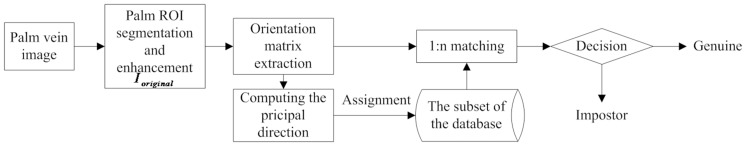
Flowchart of the system.

To solve the previously mentioned problems, this paper proposes a simple and useful classification of palm–vein identification based on the principal direction features. The flowchart of the palm–vein identification approach is shown in Fig. 1. First, the input palm–vein image undergoes pre-processing steps, including ROI extraction and nonlinear enhancement, to extract a stable ROI called 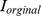, with the size of 128×128. Second, the principal direction and the orientation matrix of 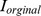 are identified. An input palm–vein image is assigned to one of the pre-specified bins based on the principal direction and is uniquely identified by one-to-one matching based on the orientation matrix. The main contributions of this paper can be summarized as follows:

1. Local features-the orientation matrix extraction: Using the continuous line-like features, the Gaussian-Radon transform is proposed to effectively determine the local orientation of each pixel in its neighborhood. The orientation matrix, which is composed of the local orientation of all pixels in the palm–vein image, is subsequently obtained.
2. Global features-the principal direction extraction: The image-pyramid, which is composed of three palm–vein images, is designed in a proportion of 2 to 1 to 0.5. At the same time, the filter-pyramid using three Gaussian-Radon filters is designed, with the same proportions as those of the image-pyramid. Finally, three orientation matrices are obtained by the image-pyramid and the Gaussian-Radon filter-pyramid, which are used to compute the principal direction of the input palm–vein image. Thus, the principal direction of the input palm–vein image can be used to classify all the palm–vein images into specific sub-classes.

The rest of this paper is organized as follows. In Method, Section 1 provides a description of the principal direction extraction. Section 2 details the palm–vein classification method. Section 3 discusses the retrieval efficiency and the response time of the classification method. Experiment results are presented and discussed followed by the conclusions.

## Method

### 1. Extraction of principal orientation features

**Figure 2:**
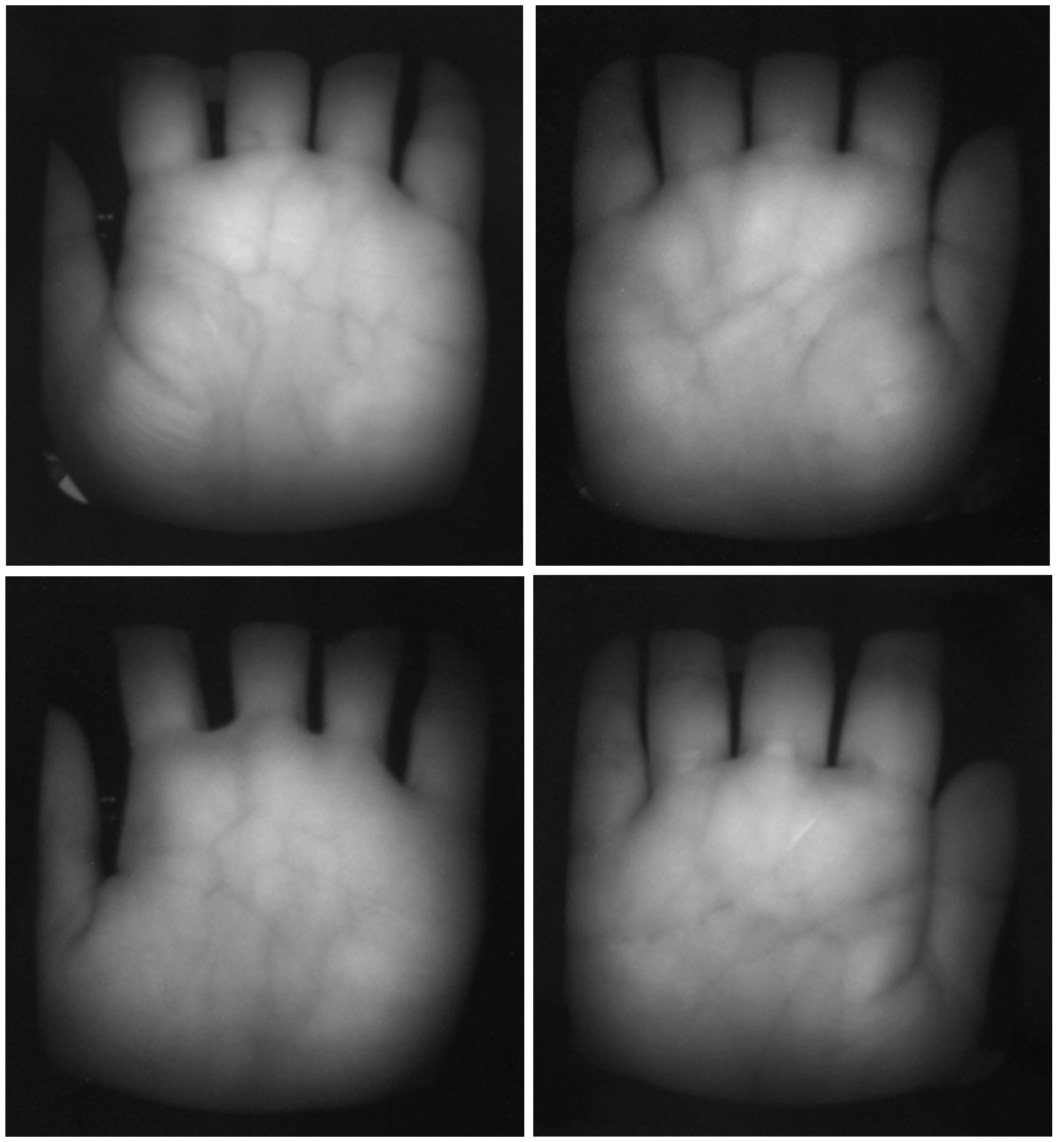
Palm–vein images in the database of this study.

Palm–vein images contain significant continuous line-like characteristics, which can be viewed as texture images. Fig. 2 shows some images in the database of this study. Thus, texture-based coding methods can be used to extract palm–vein features. Among these texture-based coding methods, the orientation-based coding is one of the efficient representation methods of palm–vein images because of its high accuracy and robust illumination, as well as fast matching [26].

**Figure 3:**
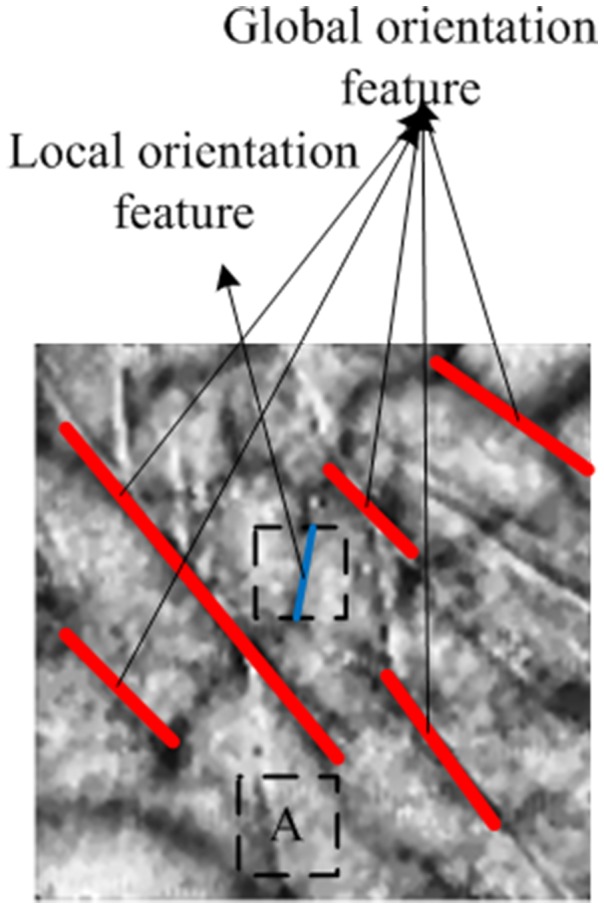
Palm–vein image.

By repeated observation, the palm–vein image is found to have two main types of orientation-based features (Fig. 3). First is the global orientation-based feature. In general, the continuous line-like features of a palm–vein image exhibit a certain tendency in the directions called as the principal direction. For example, as shown in Fig. 3, the direction of red lines comprises the majority, which is 120° in relation to the x-axis. Second is the local orientation-based feature. The veins in the neighborhood of a pixel have directions called the local orientation. The matrix composed of the local orientations of all pixels is called the orientation matrix. The principal direction of the palm–vein image is used to classify all palm–vein images into specific bins. The orientation matrix is adopted to uniquely identify the input palm–vein image by one-to-one matching with the candidates in the corresponding bin. Section 1.1 of this study introduces the extraction of the orientation matrix of the palm–vein image based on the Gaussian-Radon transform, while Section 1.2 describes the calculation of the principal direction of the palm–vein image based on the orientation matrix.

#### 1.1 Orientation matrix extraction based on the Gaussian Radon transform

**Figure 4:**
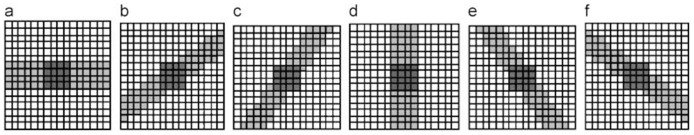
16×16 MFRAT at the directions of 0°, π/6, 2π/6, 3π/6, 4π/6, and 5π/6, in which Lk is 4 pixels wide.

According to the characteristic of the palm–vein image and based on the Radon transform [33], the literature [12], [32] proposed the Modified Finite Radon Transform (MFRAT), with the definition of 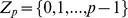, where *p* is a positive integer. The MFRAT of real function 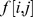 on the finite grid 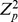 is defined as:
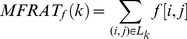
(1)where 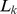 denotes the sets of points that comprise a line on the finite grid 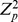:
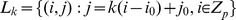
(2)where 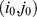 denotes the center point of 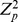, and *k* represents the corresponding slope/orientation of 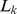. The experiments have proven that six orientations have the best results [34]. Finally, the direction of the center point 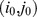 of 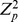 can be calculated by a winner-take-all rule:
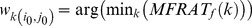
(3)where 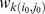 is the winner index of the orientation of center point 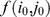. The orientations of all pixels can also be calculated if the center of lattice 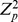 moves over an image pixel-by-pixel. Fig. 4 shows an example of MFRAT whose size is 16×16 at the directions of 0°, π/6, 2π/6, 3π/6, 4π/6, and 5π/6. In the 16×16 MFRAT, 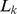 is 4 pixels wide.

The MFRAT method has the following steps. First, the MFRAT at the directions of 0°, π/6, 2π/6, 3π/6, 4π/6, and 5π/6 is designed. 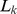 (the black and gray pixels in MFRAT as shown in Fig. 4) is assigned as the gray value of 1, and another is 0. The convolution of 6 filters and a palm–vein image is performed. Finally, the winner index of the orientation of a pixel is obtained using the winner-take-all rule, which is viewed as the local orientation of the pixel.

The MFRAT method can effectively extract finite length lines with the adjustable line-width. Thus, the MFRAT method is more suitable in extracting the orientation of the lines in the palm–vein image than the Radon method. However, MFRAT has the following limitations. First, the lattices in MFRAT 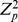 reflect a square. When the lines 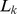 change with the orientation of *k*, the initial response of MFRAT at different directions differs. That is, the total number of pixels with the value of 1 will change at different directions. The initial conditions of the competition coding will then differ, which will influence the accuracy of the coding. For example, the initial response of MFRAT at the directions of 0° and 3π/6 is the smallest, while that of near to 45° is the largest. Second, the pixels in the lattices 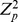 have the same contribution to the center pixel regardless of their distance from the center pixel, which will affect the coding value of the center pixel. For example, as shown in Fig. 3, when the center pixel moves to the region A, the contribution of the veins remains large regardless of their distance from the center pixel.

**Figure 5:**
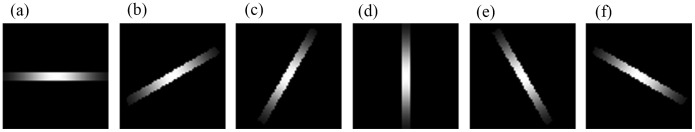
63×63 Gaussian-Radon filters at the directions of (a) 0°, (b) 30°, (c) 60°, (d) 90°, (e) 120°, and (f) 150°.

With the previously described limitations, the Gaussian-Radon transform is proposed. The Gaussian-Radon of a pixel 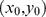 in palm–vein image *I* is defined as:
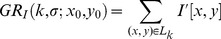
(4)where 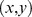 denotes the pixels in the neighborhood of 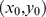. *L_k_* refers to the lines with the slope of *k*, which satisfies Equation (2). 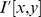 is defined as:
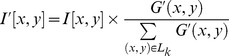
(5)where 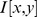 is the gray level of the pixel 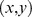 in palm-vein image *I*. And 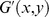 is defined as:
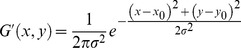
(6)where 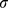 is the standard deviation of the Gaussian envelope. The weighting rules in the Gaussian function ensure that the contributions of the pixels in the neighborhood of the center point are directly proportional to the distance of the pixels to the center point. Besides, the operation of normalization makes the initial response of the Gaussian-Radon filters at different directions to be same. Fig. 5 illustrates an example of Gaussian-Radon filters, whose size is 63×63, at six directions. In the Gaussian-Radon filters, 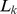 is 5 pixels wide and 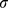 is 14.

Similarly, the convolution operation of six Gaussian-Radon filters and a palm–vein image is performed. The orientation 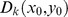 of the center point 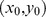 can be calculated by the winner-take-all rule, as expressed in Equation (7), which is the winner index of the orientation at the center point 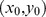. With six Gaussian-Radon filters, the value range of the orientation 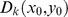 is from 1 to 6.
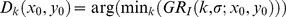
(7)


The orientation matrix (OM) is obtained by moving the center point 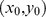 over an image pixel-by-pixel:
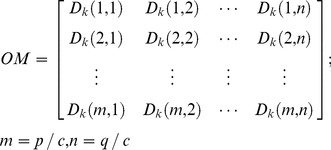
(8)where 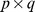 is the size of the image, 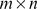 is the size of OM, and *c* denotes the line-width of the lines *L_k_*. The size of OM is inversely proportional to line width.

#### 1.2 Computing for the principal direction of the palm–vein image

**Figure 6:**
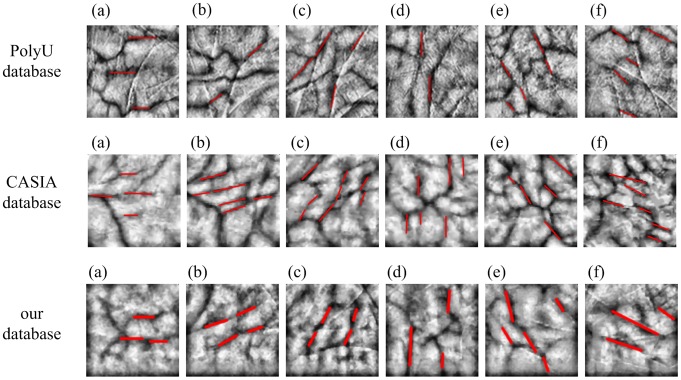
Palm–vein images at major directions of (a) 0°, (b) 30°, (c) 60°, (d) 90°, (e) 120°, and (f) 150° in PolyU, CASIA, and the database of this study.

With the irregular texture structures of the palm–vein and the low contrast of the palm–vein images, the approaches used in the fingerprint and the palm print classification methods are difficult to employ in palm–vein classification. However, a specific tendency in the directions of the continuous line-like features in a palm–vein image is discovered by repeated observation. As shown in Fig. 6(a), most of the directions of the palm–vein texture structures are horizontal, that is, 0° with respect to the x-axis. Those reflected in Figs. 6(b), 6(c), 6(d), 6(e), and 6(f) prefer the directions at 30°, 60°, 90°, 120°, and 150°, respectively. This tendency of direction is regarded as the principal direction of the palm–vein image. Principal direction features are extracted using the following algorithm:

1. The image-pyramid construction: An input palm–vein image is defined as the original image 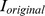, whose size is 128×128. 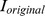 is doubled to obtain 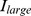 with the size of 256×256 by the bicubic interpolation, and 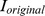 is reduced to obtain the half-size image 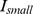, with the image size of 64×64 by down-sampling. The image-pyramid composed of 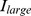, 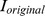, and 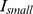 in a proportion of 2 to 1 to 0.5 is acquired.
2. The Gaussian-Radon filter-pyramid construction: Three Gaussian-Radon filters, namely, 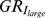, 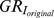 and 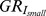, are designed, with their correspondence parameters having the same proportion as the image-pyramid.
3. Orientation matrix extraction: Three orientation matrices, namely, 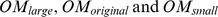 are obtained by the convolution operation of the image-pyramid and of the Gaussian-Radon filter-pyramid based on the extraction methods detailed in Section 1.1.
4. The statistical distribution of the local orientation in *OM*: Using the original palm–vein image 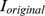 as an example, the statistical distribution of the local orientation in 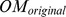 is obtained, and then the global orientation of the image 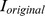 is calculated by:
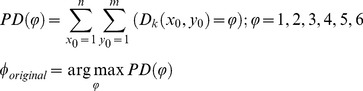
(9)where 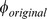 denotes the winner index of the global orientation of the image 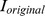, *m×n* is the original size of 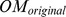, and 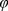 represents the possible values of 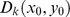, i.e., 1–6 Similarly, the winner indexes, 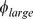 and 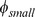, of the global orientation of the images, 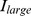 and 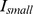, in the image-pyramid can be calculated.
5. Calculating the principal direction: Finally, the principal direction 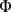 of the original palm–vein image 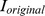 is defined as
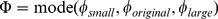
(10)where the mode operation is the most frequent value in the set [37].

Therefore, calculating the principal direction 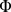 of the original palm–vein image 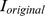 ensures the stability of the feature, regardless of noise, the contraction and relaxation of veins, and the elasticity of the palm.

### 2. Palm–vein classification

In existing literatures, the approaches for palm–vein identification assign all palm–vein images into one database. Therefore, during the identification process, the correct correspondence of an input palm–vein image is obtained by matching the input image with all the samples in the database. The searching method is called as the traditional method that demonstrates difficulty in meeting the real-time requirements of the palm–vein identification system, especially with a large database.

**Figure 7:**
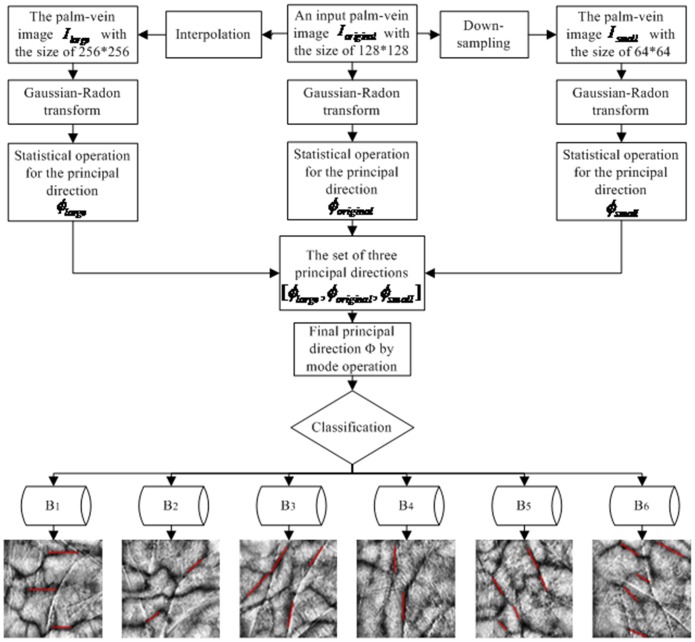
Flowchart of the sub-classes construction.

To solve identified problems, a simple and useful classification method for palm–vein identification based on the principal direction features is proposed. When used in the registration phase, the registration samples in the database are assigned into several bins. In the identification phase, the test sample is only required to match one-by-one with the samples in the corresponding bin. In this palm–vein classification approach, the number of matching for one test sample is significantly reduced, and high recognition speed is achieved, while better recognition accuracy is maintained. The classification process is shown as follows:

The principal direction 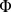 of the palm–vein image is extracted by the methods described in Section 1.2. Based on the principal direction 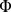, the palm–vein database (*DB*) is classified into six sub-classes expressed as 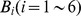. The construction of six sub-classes is shown in Fig. 7. 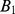 denotes the bin that contains the palm–vein images with the principal direction 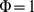. The palm–vein images in the bin 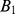 have the orientation tendency of 0°. Similarly, 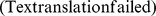 denote the bins containing images with the principal direction 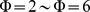. And the palm–vein images in the bins 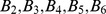 have the orientation tendency of 30°, 60°, 90°, 120° and 150°, respectively.

Because of particular characteristics of palm–vein, such as low contrast and biased information of the palm–vein image due to different collections, the principal directions of palm–vein images from the same palm may be not consistent. And palm–vein images from the same palm may have more than two principal directions. However, the characterisitics of adjacency in the principal directions of palm–vein images from the same palm is discovered by repeated experiments, that is, these palm–vein images will be assigned to the adjacent bins. Therefore, in the identification process, the searching range will be enlarged to the neighborhood bins when the true correspondence between the input and the database palm–vein image is not found in the corresponding bin.

Accuracy can clearly be traded off for efficiency. Searching beyond the top one class will provide higher accuracy but lower efficiency. To improve recognition efficiency, while maintaining better recognition accuracy, the following are formulated: (1) The search will end when the true correspondence between the input and the database palm–vein image is found. (2) If the true correspondence from the corresponding bin 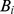 is not found, the neighborhood bins, 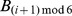 and 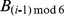, of 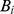 will be searched. Adjacency of the subclasses is defined based on the principal direction. For example, principal directions of 30° and 150° are the neighborhood of 0°. Therefore, the subclasses, 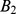 and 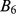, are in the neighborhood of 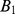. Similarly, the subclasses, 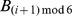 and 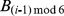, are in the neighborhood of 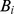.

### 3. Evaluation analysis

#### 3.1 Retrieval efficiency and retrieval accuracy

In palm–vein identification for a large database, the unique identification of the input palm–vein image is a retrieval problem. In this study, retrieval refers to filtering out the bins of candidate palm–vein patterns for finer one-to-one matching in a given input palm–vein image, until the palm–vein image is identified. In one-to-one matching, OM is regarded as the feature template and is used as the similarity measurement in [18] to compute the matching score between two feature templates. If the matching score is larger than the predefined threshold, the search ends, and the retrieved candidate reflects the true correspondence of the input image. The predefined threshold is the value around the matching score that corresponds to the EER. This threshold is determined by trial and error.

Given that the purpose of the retrieval is to find the true correspondence between the input and the database palm–vein image, retrieval accuracy and retrieval efficiency must also be considered to evaluate the proposed method.

If one of the retrieved candidates originates from the same palm as the input, the retrieval is successful for this input palm–vein image. Otherwise, the retrieval is a failure. Therefore, retrieval accuracy is calculated by the percentage of the retrieval success of input palm–vein images. The higher the retrieval accuracy, the better the performance of the system.

For one test sample, the percentage of the retrieved palm vein patterns from the database in one palm vein pattern represents the retrieval efficiency. In Section 2, we mention that the search will end when the true correspondence between the input and the database palm–vein image is found. Therefore, the number of the retrieved palm vein patterns varies with each query. Hence, in a test set, the average percentage of the retrieved palm–vein patterns from the database in all input palm–vein patterns represents retrieval efficiency. Because the retrieval efficiency is measured by the searching range, smaller searching range equates to smaller retrieval efficiency showing better performance of the system.

To demonstrate the validity of the retrieval method, 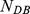 that denotes the total number of samples in the entire database and 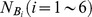 that denotes the total number of samples in bins 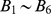 are defined. The relationship between 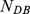 and 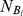 is expressed as:
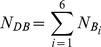
(11)

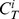
 denotes the matching number of the *i*th sample by the traditional method, and



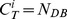
(12)With 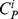 as the matching number of the *i*th sample by the proposed method, based on the rule described in Section 2, the number of retrieved candidates differs for each input sample, resulting in different values of 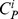 among different input samples:
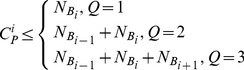
(13)where *Q* is the number of bins that must be searched, 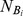 denotes the total number of samples in the corresponding bin 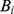, and 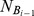 and 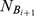 denote the total number of samples in the neighborhood bins, 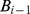 and 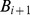, of 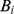. According to Equation (11), we can conclude:
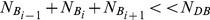
(14)in which the matching number of the search in three bins by the proposed method remains lesser than that by the traditional method.

Therefore, using Equations (12), (13) and (14), there is:
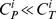
(15)


It means that the matching number of the *i*th sample by the proposed method is lesser than that by the traditional method.

Given the total number of test samples represented as *S*, the sum of matching number for *S* test samples using the traditional method and proposed method can be represented by 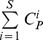 and 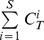, respectively. And
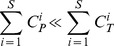
(16)


Based on the above definition of the retrieval efficiency, the retrieval efficiency of the traditional and proposed methods, 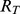 and 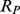, can be calculated as follows:
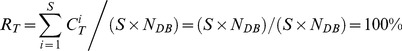
(17)




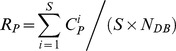
(18)


Therefore, using Equations (16), we can conclude:
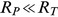
(19)


It means that the retrieval efficiency of *S* test samples by the proposed method is lesser than that by the traditional method, which shows the superiority of the proposed method (See Section 3.2 in Results and Discussion for details). If the database has millions of samples, the matching number of one test sample is significantly reduced by the proposed method, considerably improving the speed of the identification.

#### 3.2 The response time of the identification process

In the palm–vein identification process, the execution time lengthens as the number of samples in the database increases, resulting in the difficulty of meeting the requirement of the system in real-time. Whether by the traditional or the proposed method, the response time of the identification process is the sum of the duration of pre-processing, feature extraction, and one-to-many matching. We assume that 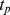, 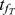, 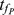 and 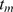 represent the response time of the pre-processing, feature extraction by traditional method, feature extraction by proposed method and one-to-one matching, respectively. Therefore, the duration of the identification process for one test sample by the traditional and proposed methods is determined as follows:

Traditional method:
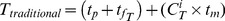
(20)


Proposed method:
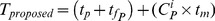
(21)where 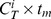 and 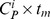 denote the time of one-to-many matching for one test sample by the traditional method and the proposed method, respectively. Obviously, the time of one-to-many matching for one test sample means that the matching number of one test sample is multiplied by the time of the one-to-one matching. Using Equations (15), we can obtain:



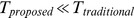
(22)The duration of the identification process is mainly restricted by the time of the one-to-many matching. If the database has millions of samples, the matching time of one test sample by the proposed method becomes shorter than that by the traditional method. (See Section 3.3 in Results and Discussion for details).

## Results and Discussion

### 1. Ethics Statements

This study was approved by the Ethics Committees of Guangdong Wicrown Information Technology Co., Ltd and Southern Medical University. Participants records/information was anonymized and de-identified prior to analysis. Therefore, the written informed consent of the participant was not obtained.

### 2. Database

Evaluation experiments are conducted on three different databases, including contact-based and non-contact databases. In this study, two contact-based databases are employed. One is the PolyU Multispectral Palmprint Database (PolyU database) [35], in which all the 6,000 images were acquired using a constrained device with finger-pegs in two sessions (six images in each session) with an average interval of nine days between the sessions. The other database is that created solely for this study, in which 1,224 images were also acquired using a constrained device but without finger- and palm-pegs. The images in the second database were captured in two sessions (six images in each session), with an average interval of thirty days between the sessions.

The non-contact database in this study is the CASIA Multi-Spectral Palmprint Database [36] in which all the 1,200 images were acquired using the non-contact device in two sessions (three images in each session), with an average interval of one month between the two sessions.

The matching of the same session data tends to achieve better matching than that of a different session because of small variations, resulting in an unreliable estimation. Therefore, the samples from the first session become the database samples, while the rest of the images become the test samples.

The experiments were conducted using Matlab 2011a in an i3-3240 CPU at 3.4 GHz with 4 GB RAM.

### 3. Evaluation experiments on the proposed classification method

The proposed classification method is evaluated by the distribution of the palm–vein images, retrieval efficiency and accuracy, and the response time of the identification process.

#### 3.1 The distribution of palm–vein images

The distribution of palm–vein images in six sub-classes in three databases by the proposed approach is shown in Table 1. The proposed classification method uniformly distributes images into sub-classes regardless of the database type. In particular, the proportions of these six categories in the PolyU database containing 6,000 samples are 19.65%, 19.13%, 17.35%, 12.60%, 16.06%, and 15.20%, which are near an even distribution.

**Table 1:** The distribution of palm–vein images in six sub-classes by the proposed method in PolyU, CASIA, and the database of this study.

DB size	B_1_	B_2_	B_3_	B_4_	B_5_	B_6_
PolyU database (6000 samples)	1179	1148	1041	756	964	912
CASIA database (1200 samples)	134	271	276	84	218	217
our database (1224 samples)	117	168	249	252	279	159

#### 3.2 Retrieval efficiency and accuracy

The predefined threshold in PolyU, CASIA, and the database used in this study are set to 0.24, 0.27, and 0.34, respectively. Comparison experiment results on retrieval efficiency and accuracy by the traditional and proposed methods are shown in Table 2. Table 2 indicates that regardless of the retrieval efficiency or accuracy, the proposed approach is outstanding in all three databases. For example, at the total number of 3,000 test samples *S* and the total number of samples in the database 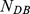 as 1500, the sum of matching number by the traditional method is 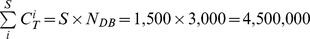, while by the proposed method is 643,377. The retrieval efficiency is 
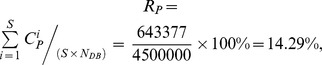
 and the retrieval accuracy is 96.67%. Besides, retrieval efficiency is 14.50% and 14.28% in the other two databases, with retrieval accuracy of 96.00% and 97.71%, respectively. Thus, the proposed method is superior in the identification process.

**Table 2:** Comparison between traditional and proposed methods in three different databases.

	PolyU database	CASIA database	our database
The total number of samples in the database *N_DB_*	1500	600	306
The total number of test samples *S*	3000	600	612
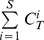 The sum of matching number by traditional method	4500000	360000	187272
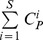 The sum of matching number by proposed method	643377	52216	26745
Retrieval efficiency *R_p_* (%)	14.29	14.50	14.28
Retrieval accuracy (%)	96.67	96.00	97.71

Methods for classifying palm veins are currently unavailable. Regarding our classification method, this approach is suitable for other state-of-the-art coding methods for extracting orientation features, such as competitive code [41], Radon transform, and Gaussian transform. We perform comparative experiments by using different coding methods with the same classification process discussed in Sections 1.2 and 2 in Method. Table 3 presents the comparison among the four coding methods in three databases with the same condition as Table 2. This table indicates that the proposed approach provides the best results for the three databases regardless of retrieval efficiency or accuracy.

**Table 3:** Performance of palm vein classification via different coding methods using PolyU, CASIA, and the database for this study.

		PolyU database	CASIA database	Our database
The sum of matching number	**Proposed method**	**643377**	**52216**	**26745**
	Competitive code	907182	61482	30798
	Radon transform	920213	65460	32870
	Gaussian transform	1022228	87813	45488
Retrieval efficiency *R_p_* (%)	**Proposed method**	**14.29**	**14.50**	**14.28**
	Competitive code	20.15	17.08	16.44
	Radon transform	20.45	18.18	17.55
	Gaussian transform	22.71	24.39	24.29
Retrieval accuracy (%)	**Proposed method**	**97.67**	**96.00**	**97.71**
	Competitive code	91.37	94.67	94.28
	Radon transform	94.77	91.67	96.90
	Gaussian transform	95.17	92.67	96.41

#### 3.3 The response time of the identification process

We assume that the feature extraction process via the traditional method uses Gaussian-Radon transform to extract the orientation features of a palm vein image that measures 128×128. Different coding methods have the ROI image and OM with the same size in three databases. Therefore, the execution times for preprocessing and one-to-one matching are approximately the same, whereas that for feature extraction is different, as shown in Tables 4 and 5. The execution time for matching will lengthen considerably as the number of samples in the database increases. The traditional method may not be able to meet the speed requirements of the palm–vein identification system, especially with a very large database.

**Table 4:** Execution time of pre-processing and one-to-one matching by different coding methods.

Operations	Pre-processing *t_p_*	One-to-one matching *t_m_*
Times(ms)	520	1.8

**Table 5:** Execution time of feature extraction by different coding methods.

	ProposedMethod	TraditionalMethod	CompetitiveCode	RadonTransform	GaussianTransform
The response time of feature extraction(ms)	56.3	40	530	58	262

The matching number is proportional to retrieval efficiency. Hence, the matching numbers from the proposed method, competitive code, Radon transform, and Gaussian transform can be reduced roughly by a factor of 6.96 (100%/14.36%, where 14.36% is the mean retrieval efficiency value in the three databases), 5.59 (100%/17.89%), 5.34 (100%/18.73%), and 4.20 (100%/23.80%), respectively, based on the retrieval efficiency results obtained via the different coding methods listed in Table 3.

**Figure 8:**
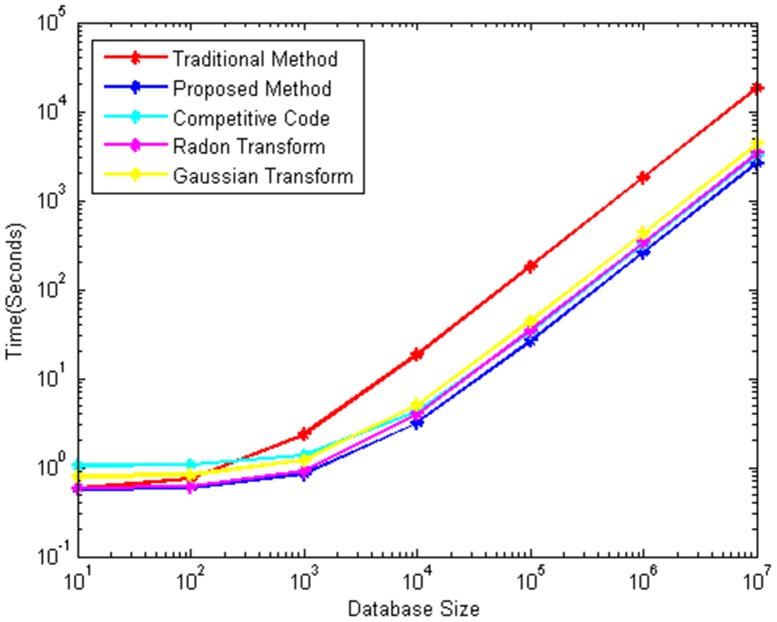
The response time of identification process for one test sample by different coding methods at different database sizes.

The identification time for one testing sample via different coding methods in a large database can be calculated using Equations (20) and (21) and the computation time listed in Tables 4 and 5. Fig. 8 shows the response time of the identification process for one test sample via different coding methods and the traditional method at different database sizes. The proposed approach is evidently more efficient than the traditional method for large databases. With 10,000 training samples in the database, the execution times for the identification process via the traditional method, competitive code, Radon transform, and Gaussian transform are 18.56, 4.27, 3.95, and 5.07 s, respectively. And the proposed approach only requires 3.16 s.

### 4. Evaluation experiments on the one-to-one matching algorithm

To verify the effectiveness of the Gaussian-Radon transform in one-to-one matching algorithm, evaluation experiments are performed on three databases. The capability to achieve high performance using a small number of registration samples is highly desirable in any biometrics system [38]. In the palm–vein recognition algorithms perspective, the Neighborhood Matching Radon Transform (NMRT) [12] demonstrates the best results. Therefore, in this study, only the comparison experiments between the proposed method and NMRT using one registration sample are considered, as shown in Tables 6–8. The results confirm the superiority of the proposed method.

**Table 6:** The rank-1 identification rate [38] and EER by different methods using the PolyU database.

Method	Gaussian-Radon Transform	NMRT [^12^]
EER	0.14%	0.21%
Rank one identification rate	99.83%	99.67%

**Table 7:** The rank-1 identification rate and EER by different methods using the CASIA database.

Method	Gaussian-Radon Transform	NMRT [^12^]
EER	0.67%	1.37%
Rank one identification rate	99.50%	96.83%

**Table 8:** The rank-1 identification rate and EER by different methods using the database of this study.

Method	Gaussian-Radon Transform	NMRT [^12^]
EER	0.09%	0.49%
Rank one identification rate	99.91%	99.35%

## Conclusions

To solve the problem of a long response time in palm–vein identification in a large database, this paper proposed a simple and useful classification based on the principal direction features. Gaussian-Radon transform was employed to extract the orientation matrix and compute the principal direction of the image. Using the principal direction as the classification index, the large database is categorized into six bins. In the identification process, the input palm–vein image was first assigned to one of the bins and then matched with the candidates in the bin one-by-one. Besides, the neighborhood rule to speed the searching process was adopted, while maintaining a relatively high accuracy. Compared with traditional methods, experiments in the three databases by the proposed method showed its advantages on retrieval efficiency and identification time, especially for large palm–vein databases.
