# Correction: Palm-Vein Classification Based on Principal Orientation Features

**DOI:** 10.1371/journal.pone.0116446

**Published:** 2014-12-19

**Authors:** The PLOS ONE Staff

The second author’s affiliation is incorrect. Yaqin Liu is not affiliated with #2 but with #1 School of biomedical engineering, Southern Medical University, Guangzhou 510515, China.
